# Low-Cost Protocol for Quantitative Measurement of *Streptococcus salivarius* in Human Saliva

**DOI:** 10.3390/life15111695

**Published:** 2025-10-31

**Authors:** Flavia-Cristina Al-Akel, Lacramioara Eliza Chiperi, Krisztina Eszter Vas, Edit Szekely, Claudia Raluca Mariean, Corina Eugenia Budin, Anca Bacarea

**Affiliations:** 1 Physiopathology Department, George Emil Palade University of Medicine, Pharmacy, Science and Technology, 540142 Targu Mures, Romania; alakelcristina@gmail.com (F.-C.A.-A.); raluca.mariean@umfst.ro (C.R.M.); corina.budin@umfst.ro (C.E.B.); anca.bacarea@umfst.ro (A.B.); 2 Doctoral School of Medicine and Pharmacy, George Emil Palade University of Medicine, Pharmacy, Science and Technology, 540142 Targu Mures, Romania; 3 Department of Pediatric Cardiology, Emergency Institute for Cardiovascular Diseases and Heart Transplant, 540136 Targu Mures, Romania; 4 Department of Laboratory Medicine, County Emergency Hospital, 530173 Miercurea Ciuc, Romania; krisztinaesztervas@gmail.com; 5 Microbiology Department, George Emil Palade University of Medicine, Pharmacy, Science and Technology, 540142 Targu Mures, Romania; edit.szekely@umfst.ro; 6 Medical Microbiology Laboratory, Targu Mures County Emergency Hospital, 540042 Targu Mures, Romania; 7 Department of Radiology, Targu Mures County Emergency Hospital, 540136 Targu Mures, Romania; 8 Pulmonology Clinic, Mures Clinical County Hospital, 540103 Targu Mures, Romania; 9 Department of Laboratory Medicine, Emergency County Hospital, 540136 Targu Mures, Romania

**Keywords:** *Streptococcus salivarius*, saliva, oral microbiota, CFU quantification, low-cost microbiology, selective culture

## Abstract

*Streptococcus salivarius* (*S. salivarius*) is a prominent oral commensal bacterium with probiotic potential and relevance to both oral and systemic health. Accurate and accessible methods for quantitative measurement of this species are needed to support microbiota studies and clinical interventions. We describe a simple, low-cost, culture-based method for quantifying *S. salivarius* in human saliva using Mitis-*Salivarius* Agar. Saliva samples from 18 healthy adult volunteers were analyzed through serial dilutions and selective plating. CFU (colony forming unit)/mL were calculated after 24 and 48 h incubation. The method proved reliable for quantifying *S. salivarius* in concentrations ranging from 5.8 × 10^5^ to 6.1 × 10^8^ CFU/mL. Although Mitis-*Salivarius* Agar is a standard medium, we demonstrate its systematic validation and optimization for human saliva in a low-resource clinical setting, where molecular tools are often unavailable.

## 1. Introduction

The study of commensal bacteria is rapidly gaining traction in the context of precision medicine, with *Streptococcus salivarius* (*S. salivarius*) emerging as a key player in maintaining oral and systemic microbial equilibrium [1]. Far from being a passive inhabitant, *S. salivarius* actively contributes to immune modulation, pathogen inhibition, and mucosal integrity, making it both a sentinel and a potential therapeutic agent [2]. As one of the predominant and generally benign members of the oral microbiota, *S. salivarius* is well known for its ability to inhibit the growth of pathogenic microorganisms through competitive exclusion and production of antimicrobial compounds [3].

Nevertheless, not all *S. salivarius* strains exhibit beneficial properties. While many are protective, certain strains can act as opportunistic pathogens, causing rare but documented cases of bacteremia and infective endocarditis, particularly in immunocompromised individuals or in those with structural heart disease [4,5]. On the other hand, several strains have demonstrated probiotic potential. For example, strain M18 has been associated with reduced plaque accumulation, dental caries, and black stains on teeth; strain K12 has been shown to lower the incidence of streptococcal pharyngitis and may also decrease the risk of viral pharyngitis, tracheitis, rhinitis, influenza, laryngitis, acute otitis media, and enteritis; and strain 24SMB has been implicated in reducing the recurrence of acute otitis media [6,7].

Assessing *S. salivarius* abundance therefore provides valuable insight into the ecological balance of the oral microbiome. Dysbiosis—an imbalance between beneficial and pathogenic microbes—has been implicated in both oral and respiratory diseases [8,9]. Monitoring *S. salivarius* levels may facilitate early detection of microbial imbalance and guide timely interventions such as probiotic therapy or antimicrobial treatment [10]. Beyond oral health, *S. salivarius* is increasingly being investigated for its systemic impact, with recent studies proposing its role as a biomarker in liver fibrosis, cardiovascular risk, and inflammatory disorders [11].

Although generally regarded as commensal, shifts in the abundance or activity of oral bacteria can influence the development of respiratory infections, cardiovascular disease, and metabolic disorders such as diabetes [12]. Thus, the presence of *S. salivarius* does not always equate to “no worries”, as it can occasionally cause bacteremia and, rarely, infective endocarditis with serious complications [2]. In addition to this rare scenario, in patients with congenital heart disease there are frequent postoperative contexts—especially following extracorporeal circulation—where quantifying the oral microbiota, and particularly *S. salivarius*, may prove useful for prophylactic strategies and potential therapeutic interventions [3].

Despite growing interest in the clinical applications of *S. salivarius*, most studies have focused on molecular quantification techniques such as qPCR or digital PCR, which, while sensitive, require expensive reagents, equipment, and trained personnel [13,14]. This limits their accessibility in routine practice, particularly in low-resource settings, field studies, or during preliminary screening phases. Therefore, developing a low-cost, culture-based method that maintains specificity and reproducibility could bridge the current gap between advanced diagnostics and day-to-day clinical or epidemiological needs.

Although Mitis-*Salivarius* Agar–based quantification of oral streptococci is an established microbiological practice, few reports have provided a low-resource protocol specifically optimized for human saliva in routine clinical laboratories outside high-income settings. 

Our work addresses this gap by (i) simplifying the workflow to basic equipment available in most district-level microbiology labs, (ii) demonstrating reproducibility with pilot-tested adjustments to dilution range and incubation time, and (iii) verifying species identity with MALDI-TOF/VITEK to ensure true *S. salivarius* counts. This contextual validation provides an evidence-based, cost-transparent alternative to proprietary rapid kits and molecular assays, especially for resource-limited environments.

## 2. Materials and Methods

### 2.1. Study Design and Population

This study is part of a broader research initiative focused on *S. salivarius*, specifically its detection and quantification using low-cost, culture-based microbiological methods. The study aimed to establish a reliable protocol for measuring *S. salivarius* levels in human saliva without the use of molecular or genetic techniques.

Eighteen apparently healthy (no cephalic/cervical inflammatory signs, no clinical infection, and no recent history of upper respiratory tract infection) adult volunteers aged between 20 and 40 years were recruited. Inclusion criteria required subjects to be free of acute or chronic oral or respiratory illness. 

Additional oral and behavioral factors known to influence salivary CFU levels were also considered. Active smokers and subjects with recent dental procedures (within the past 2 weeks) or antiseptic mouthwash use (e.g., chlorhexidine within 48 h) were excluded. 

Exclusion criteria also included antibiotic or probiotic use within the previous two weeks, and ingestion of food/drink or toothbrushing less than two hours before saliva collection. Clinical and anamnestic data were recorded using a standardized questionnaire. Informed consent was obtained from all subjects involved in the study. 

This study should be understood as a methods validation/pilot feasibility study, not as an attempt to define normative reference ranges. The sample size (N = 18 healthy adults aged 20–40 years) was pragmatically chosen to assess reproducibility and paired differences (24 h vs. 48 h readings), and to generate preliminary estimates to power future larger-scale validation studies.

### 2.2. Sample Collection and Handling

Participants provided unstimulated saliva samples collected in sterile, wide-mouth containers. Samples were transported at room temperature immediately (<30 min from collection to plating). Approximately 2 mL of saliva was obtained per subject.

Saliva samples were collected and processed manually, using standard microbiological techniques, with minor adjustments to sample volume and dilution to optimize reproducibility. This simple protocol allowed *S. salivarius* quantification without specialized equipment.

### 2.3. Culture Medium Preparation and Selectivity

The selective medium used for this study was based on a formulation obtained from international laboratories, not previously applied in Romanian microbiological research. The Mitis-*Salivarius* Agar (MSA, Liofilchem, Roseto degli Abruzzi, Italy) enriched with potassium tellurite 1% was prepared according to the manufacturer’s indication: 90.0 g of powder was suspended in 1 L of distilled water and boiled until completely dissolved. After sterilization at 121 °C for 15 min the culture media was left to cool up to 45–50 °C. Once cooled down 1 mL of potassium tellurite 1% was added aseptically and the media was poured in Petri dishes. The prepared plates were stored at 8 °C, sterility and growth control was performed in-house, using the reference *S. salivarius* ATCC 13419 (Sanimed, Bucharest, Romania) strain for positive control and *Escherichia coli* ATCC 25922 (ThermoScientific^TM^, Waltham, MA, USA) strain for negative control. Although *Escherichia coli* is phylogenetically distant from oral streptococci, it was selected as the negative control in accordance with the official Mitis-*Salivarius* Agar (Liofilchem) manufacturer’s recommendations. 

This culture medium supports the selective growth of *S. salivarius* and related streptococci. Initial pilot testing involved cultivating a pure *S. salivarius* reference strain to establish characteristic colony morphology (blue, smooth or rough “gum drop” colonies, 1–5 mm in diameter—M-type colonies) and growth pattern under defined conditions. 

### 2.4. Serial Dilution and Culturing Protocol

Each saliva sample was serially diluted in sterile phosphate-buffered saline (PBS) to prepare 10^−5^, 10^−6^, and 10^−7^ dilutions. According to the manufacturer’s technical specifications for Mitis-*Salivarius* Agar (Liofilchem), the recommended inoculum for productivity testing is 50–100 CFU, while the inoculum range for selectivity testing is 10^4^–10^6^ CFU. These values provide a practical reference for the expected detection and quantification range under standard laboratory conditions. 

From each dilution, 100 µL aliquots were plated in duplicate on MSA plates using aseptic techniques. All plates were incubated in a CellXpert CO_2_ incubator (Eppendorf, Hamburg, Germany) at 35 °C in a 5% CO_2_ atmosphere, with controlled humidity to prevent condensation that could affect colony morphology on Mitis-*Salivarius* Agar. Temperature and CO_2_ concentration were checked daily by trained personnel, and all values were automatically recorded in the device log.

The incubation temperature of 35 °C was selected in accordance with the manufacturer’s instructions for Mitis-*Salivarius* Agar (Liofilchem). This temperature provides optimal colony morphology and differentiation in most clinical laboratories, although slight variations (35–37 °C) are acceptable depending on local standard procedures.

### 2.5. Colony Identification and Confirmation

The aim of the pilot study was the visual accommodation with the colony morphology, as well. While plating the saliva samples, beside M-type colonies, we noted the presence of occasional small, flat colonies. MALDI-TOF mass spectrometry (EXS2600, Zybio, Chongqing, China) and VITEK 2 Compact (bioMérieux, Marcy-l’Étoile, France) were used to identify the different colony types. Several colonies were picked, among the two colony types. All the small, flat colonies were identified as *S. mitis* and the M-type colonies were *S. salivarius.* During the quantification stage the colony growth was assessed visually. The small, flat colonies, consistent with other oral streptococci were excluded from *S. salivarius* counts. 

During the quantification phase, at least one MALDI-TOF confirmation was performed per sample to ensure species identity, following ≥2 confirmations per sample during the pilot phase used to establish colony morphology reference criteria.

### 2.6. Quantification of CFU/mL

Colony counts were performed manually, without the use of image analysis software. Rarely, partial colony confluence (“spreader” or heaped/mucoid colonies) was observed after 24 h of incubation. These colonies were initially distinguishable and were therefore counted according to their individual outlines observed at 24 h, even if they became confluent by 48 h. 

For each sample, CFU/mL were calculated based on the dilution level and the number of colonies counted on plates were clearly visible (typically at 48 h readings). Only countable plates (30–200 colonies) were used to derive CFU/mL values—no extrapolations were made from TNTC values. Plates with >200 colonies were excluded from calculation. The formula used for calculation was:

CFU/mL = (Number of colonies × Dilution factor)/mL.

After obtaining the CFU count for each dilution, the average CFU/mL was calculated and used for further interpretation. The averaging approach was applied across duplicate plates per dilution, as detailed in Table 1, to minimize inter-plate variability and ensure consistent quantification.

### 2.7. Storage Impact Assessment

For the storage impact assessment, eight saliva samples were plated and quantified immediately after collection to establish the initial CFU/mL values of *S. salivarius*. Subsequently, four samples were stored at room temperature (approximately 22–24 °C) and four additional samples (unpaired) were stored under refrigeration at 4 °C for 24 h. This design was selected to evaluate the influence of short-term storage on bacterial viability in scenarios where immediate plating after collection might not be feasible, as recommended by the protocol. After storage, samples were re-plated following the same serial dilution protocol. Colony counts were compared to initial results to assess potential loss or proliferation of *S. salivarius*.

All data were documented systematically, and colony counts were independently verified by two trained observers to ensure consistency and reliability.

## 3. Results

A total of 18 saliva samples were successfully processed. Colony counts of *S. salivarius* varied significantly among individuals. The range of CFU/mL detected after 48 h incubation was between 5.8 × 10^5^ and 6.1 × 10^8^ CFU/mL. Generally, colonies were more numerous and more easily visible after 48 h compared to 24 h of incubation.

### 3.1. Colony Growth at 24 h vs. 48 h of Incubation

**Figure 1:**
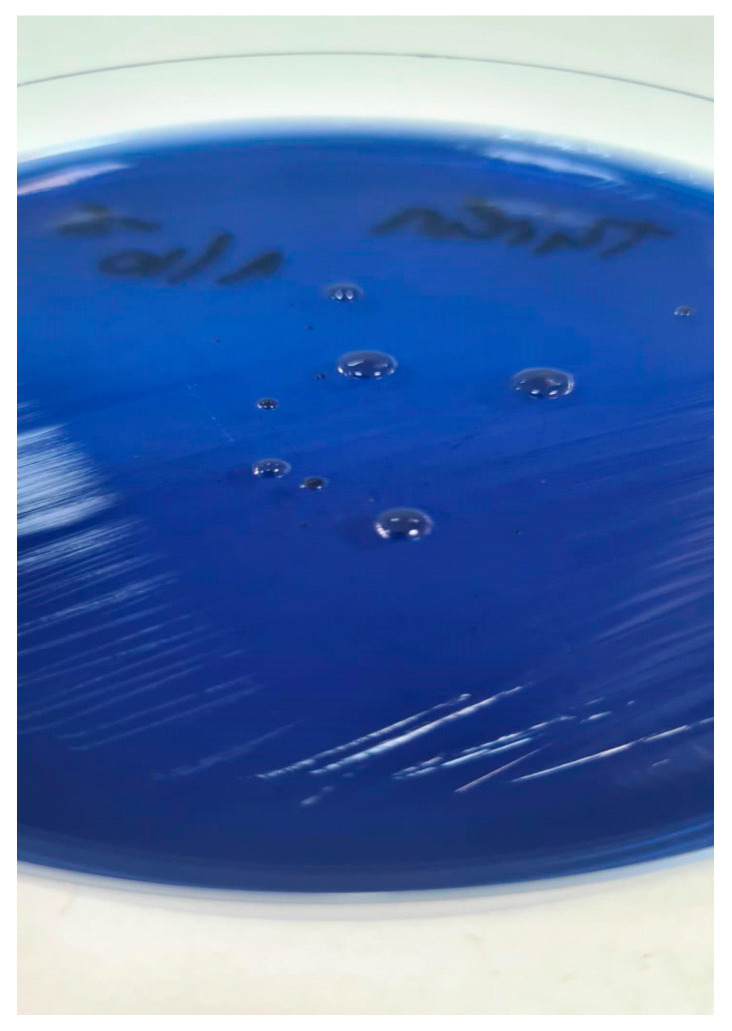
Colony morphology of *S. salivarius*.

*S. salivarius* forms blue, gum-drop-like, M-type colonies (volcano-shaped, ‘gum-drop’ morphology) on MSA after 24 h incubation at 35 °C in a 5% CO_2_ atmosphere, which is more evident after 48 h incubation, as shown in Figure 1. The small colonies were identified as *S. mitis* and the bigger, mucoid colonies proved to be the *S. salivarius*.

Across the 18 samples, rare colonies on primary plates displayed non-typical morphology; MALDI-TOF/VITEK analysis confirmed these to be non-*S. salivarius* (primarily *S. mitis*/*vestibularis*). These colonies were not included in CFU calculations, supporting the need for confirmatory testing.

Table 2 shows the comparison of CFU/mL for each of the 18 samples after 24 and 48 h of incubation.

Most samples showed either a small or moderate increase in colony counts after 48 h. Only one sample (Sample 3) showed a slight decrease in colony count between 24 h and 48 h. The same plates were reassessed at both incubation time points (24 h and 48 h) to ensure within-sample consistency. The minor decrease observed in Sample 3 was therefore interpreted as a possible effect of partial autolysis during extended incubation rather than inter-sample variation.

**Figure 2:**
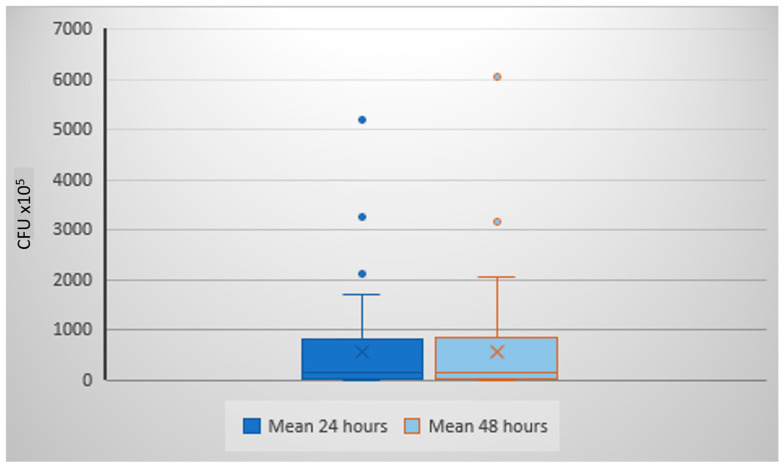
Boxplot analysis of CFU/mL at 24 and 48 h of incubation.

**Figure 3:**
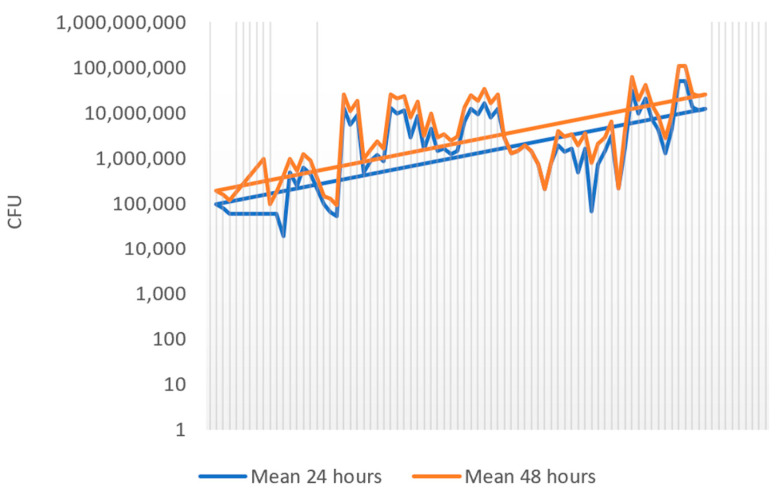
Scatter plot analysis of CFU/mL at 24 and 48 h of incubation.

The mean *S. salivarius* count at 24 h was 542.7 × 10^5^ CFU/mL (DS ± 975.1 × 10^5^; median 150 × 10^5^), while at 48 h it increased to 645.1 × 10^5^ CFU/mL (SD ± 1141.9 × 10^5^; median 162.5 × 10^5^). The difference was statistically significant (*p* < 0.0001, Wilcoxon matched pairs test), confirming that 48 h incubation provides more accurate colony detection. Boxplot analysis (Figure 2) illustrates the wide inter-individual variability. A scatter plot (Figure 3) shows the consistent trend of increased colony counts after extended incubation.

An F-test for equality of variances between the two groups showed no statistically significant difference (F = 0.799, df = 74, 74, *p* = 0.168). Thus, the assumption of homogeneity of variances was satisfied.

### 3.2. Effect of Sample Preservation on S. salivarius Viability

To assess the effect of short-term storage, saliva samples were re-plated after 24 h preservation at room temperature (RT) or at 4 °C. Table 3 presents the CFU/mL values for eight samples tested. Compared to the initial plating, CFU/mL counts showed a mean increase of 182.5 × 10^5^ CFU/mL at room temperature and 238.3 × 10^5^ CFU/mL under refrigeration. However, two samples demonstrated a reduction in CFU/mL (decrease of 75% and 100%), suggesting possible strain-specific autolysis or technical inconsistencies. Overall, bacterial viability tended to be better preserved under refrigeration than at room temperature, but immediate processing yielded the most reliable counts.

## 4. Discussion

### 4.1. Role of S. salivarius in Health and Disease

A recent systematic review [7] consolidated the available evidence on the use of *S. salivarius* probiotics as a prophylactic intervention in pediatric populations for otic and respiratory tract infections, as well as other infectious conditions and oral health. *S. salivarius* is efficient in the prevention of acute otitis media, pharyngitis, tonsillitis, adenoiditis, SARS-CoV-2 and other viral infections, periodic fever, aphthous stomatitis, pharyngitis and adenitis syndrome episodes, as well as various oral and dental disorders [6].

In the orthodontic field, Al-Melh et al. found significantly elevated salivary levels of *S. salivarius*—as measured by real-time PCR—in patients who had been wearing fixed orthodontic brackets for 12 months compared to controls, implicating bracket placement in shifts toward cariogenic bacterial populations [14]. 

Recent advances in oral microbiome research have emphasized the importance of *S. salivarius* as a biomarker and modulator of systemic health [10]. Its presence and balance are increasingly being studied in relation to cardiovascular diseases, metabolic disorders, and even mental health conditions, through the oral–gut–brain axis [6]. Moreover, Iwasaki et al., using digital PCR to quantify *S. salivarius* in fecal samples, highlights its emerging role as a non-invasive biomarker for liver fibrosis [13].

Animal studies also support its probiotic potential; for instance, pretreatment with *S. salivarius* K12 in mice exposed to *Mycoplasma pneumoniae* infection resulted in significant reductions in pulmonary inflammation, decreased pathogen gene expression, and attenuated lung injury [15]. Additionally, *S. salivarius* K12 has been shown to alleviate radiation-induced oral mucositis and rebalance oral microbiota in mice, suggesting therapeutic potential for cancer patients undergoing radiotherapy [16].

### 4.2. Validation of the Method

This study establishes a culture-based protocol that enables reliable and cost-effective quantification of *S. salivarius* in human saliva. Utilizing MSA with a 48 h incubation period, the method provided clear visualization of colonies, markedly improving counting precision compared to 24 h incubation. While commercial molecular kits are available, they often present challenges in accessibility and affordability, especially in low-resource environments. The protocol presented here offers a pragmatic alternative for both clinical applications and field research in oral microbiome surveillance.

Minor modifications to inoculum volume, dilution ranges, and incubation time were introduced after pilot testing to improve reproducibility and inter-observer agreement, which to our knowledge have not been reported for this specific application in human saliva.

One of the key insights from our results is the necessity of extended incubation time. Colonies of *S. salivarius* were often faint or not fully visible at 24 h. However, at 48 h, colony morphology became distinct—blue, gum-drop-like M-type colonies on Mitis-*Salivarius* Agar—which improved count accuracy and reduced the risk of underestimation. 

It is also important to consider that saliva samples may occasionally lack *S. salivarius* altogether or may contain phenotypically atypical or divergent strains that do not conform to classical colony morphology. Such variants could escape visual detection in purely culture-based workflows, underscoring the value of complementary molecular confirmation in future studies. 

The statistically significant increase in CFU/mL after 48 h further supports the necessity of longer incubation as a standard in future protocols using culture-based method.

The F-test demonstrated no significant difference in variance between the two incubation time points (F = 0.799, *p* = 0.168). This finding indicates that the dispersion of *S. salivarius* colony counts was comparable in both conditions, suggesting that the observed increase in mean values after 48 h incubation reflects a consistent trend rather than variability-driven fluctuations. The result strengthens the robustness of comparative analysis, supporting the conclusion that extended incubation yields more reliable and reproducible quantification.

Marked inter-individual variability was observed across saliva samples, with CFU-counts ranging over more than two orders of magnitude—from 5.75 × 10^5^ to 6.05 × 10^7^ CFU/mL—suggesting significant heterogeneity in oral microbiota even among healthy individuals. 

The protocol’s reproducibility was substantiated via duplicate plating and identity confirmation using MALDI-TOF and the VITEK 2 Compact system. Refrigeration at 4 °C was shown to preserve bacterial viability better than room temperature; however, immediate processing remained the gold standard to minimize variability and risk of underestimation due to potential autolysin-related viability loss in certain strains [17,18,19].

Culture-based enumeration of *S. salivarius* is not itself novel; however, the present study fills a practical implementation gap. We optimized and documented a reproducible protocol that can be performed in standard hospital laboratories without molecular infrastructure. Key differentiators include the following: (1) workflow simplification—minor but critical modifications to sample volume, dilution factors, and mandatory 48 h incubation established through pilot testing; (2) local validation—systematic testing in a Romanian clinical setting where molecular diagnostics are often inaccessible; and (3) cost-effectiveness with species-level confirmation—integration of MALDI-TOF/VITEK confirmation to overcome the specificity limitations of traditional plating. These elements distinguish our method from existing culture-based descriptions and highlight its suitability for large-scale surveillance or perioperative monitoring where commercial dip-slide kits or molecular assays may be impractical.

### 4.3. Comparison with Molecular Quantification Techniques

In comparison to existing molecular techniques, the culture-based protocol is different in terms of accessibility and practicality. Quantitative PCR (qPCR) is widely regarded as the gold standard for bacterial quantification due to its high sensitivity and specificity, often detecting as few as 10–100 copies of bacterial DNA within a few hours [13,14,20]. However, qPCR requires expensive reagents, thermocyclers, and trained personnel, which may not be feasible in low-resource or field settings. Digital PCR (dPCR) offers even greater accuracy and absolute quantification without the need for standard curves, but the associated costs are considerably higher, and throughput is limited [13,21,22]. Loop-mediated isothermal amplification (LAMP) presents a faster and simpler alternative, capable of amplifying DNA under isothermal conditions with minimal equipment, making it attractive for forensic or rapid diagnostic applications [19,23]. Nevertheless, LAMP assays still require DNA extraction, primers with high specificity, and fluorescence or turbidity monitoring devices, which can increase costs and technical complexity.

By contrast, the culture-based method requires only basic microbiology equipment, Mitis-*Salivarius* Agar, and a standard incubator, making it substantially more affordable and scalable for routine use. While less sensitive than molecular techniques and slower due to the 48 h incubation period, it provides viable bacterial counts expressed as CFU/mL, which remain the most clinically interpretable units in microbiology. 

Table 4 provides a comparative overview of the major methods, highlighting trade-offs in sensitivity, cost, turnaround time, and applicability.

### 4.4. Clinical and Practical Implications

The findings of this study have several clinical implications for vulnerable populations, particularly individuals with congenital heart disease (CHD), immunocompromised patients, and those undergoing major surgery. 

In children with CHD, multiple studies have reported significant alterations in oral microbiota composition compared to healthy controls, including an overrepresentation of cariogenic and periodontal pathogens, which may predispose to bacteremia and infective endocarditis [24]. In adults with CHD or congenital valve anomalies, the reduction in antibiotic prophylaxis (fewer indications) following guideline updates has been associated with an increased incidence of oral streptococcal infective endocarditis, underlining the importance of microbial monitoring and preventive oral health strategies in this group [25].

The perioperative period represents another high-risk context in which oral microbiota monitoring may be clinically valuable. Patients undergoing cardiac valve surgery, particularly those requiring intubation, demonstrate significant increases in oral bacterial counts immediately after surgery, suggesting that oral streptococci can contribute to postoperative infection risk [26]. 

Similarly, immunocompromised populations may experience rapid microbiota shifts, where expansion of opportunistic streptococci may serve as an early marker of systemic infectious susceptibility.

Taken together, these observations highlight the potential of a simple, culture-based quantification method for *S. salivarius* as a complementary tool in clinical surveillance. By providing accessible and viable bacterial counts, this protocol could be integrated into perioperative care pathways, CHD follow-up programs, and immunocompromised patient monitoring to support both preventive and therapeutic decision-making [27].

### 4.5. Advantages, Limitations and Future Directions

Advantages of the proposed method are cost-effectiveness, simplicity, minimal equipment, applicability in routine settings. 

Despite its strengths, the culture-based approach also carries intrinsic limitations. One important aspect is that Mitis-*Salivarius* Agar (MSA), while selective, is not fully specific for *S. salivarius*. Other oral streptococci, such as *S. mitis* or *S. vestibularis*, can occasionally produce colonies with overlapping morphology, which may lead to misclassification, particularly in the absence of confirmatory tests [28,29] because colony morphology is observer-dependent. Even with modern identification techniques such as MALDI-TOF MS, there remains a small to medium risk of misidentification due to limitations in spectral databases and the close phylogenetic relatedness of oral streptococci [30]. These issues highlight the need for careful interpretation of culture results and underscore the complementary role of molecular methods when higher taxonomic resolution is required. Inter-operator variability in plating technique may also contribute to inconsistencies.

In this context, it is important to clarify that our study was not designed as a molecular validation. While the protocol was systematically optimized and cross-checked by MALDI-TOF and VITEK confirmation, we recognize that a “rigorous validation” in the molecular sense would require parallel testing using qPCR or whole-genome sequencing (WGS) as gold-standard methods. We have therefore deliberately avoided overstating the validation scope and defined our approach as a low-cost, semi-quantitative culture-based optimization suitable for laboratories lacking molecular infrastructure. In line with Abdelbary et al. [31], we also acknowledge that MALDI-TOF may occasionally misidentify closely related viridans streptococci, and that WGS remains the most accurate method for species-level discrimination.

Another methodological limitation concerns the choice of *Escherichia coli* as a negative control. While *E. coli* is phylogenetically distant from oral streptococci, it was used according to the official Mitis-*Salivarius* Agar (Liofilchem) protocol, which specifies this strain for quality control. This ensured procedural standardization and reproducibility; however, future work could benefit from including a more closely related non-target oral streptococcal species, such as *S. mitis* or *S. vestibularis*, to provide a stricter assessment of medium specificity.

In addition, the possibility of partial autolysis in certain strains—such as that observed in one sample showing reduced CFU counts after 48 h—should be considered when interpreting time-dependent quantification results.

Another limitation of this work is the small, homogeneous cohort of only 18 healthy young adults. Although it allowed careful methodological testing, the restricted sample size and limited demographic diversity preclude generalization to broader populations. We did not collect or analyze detailed clinical, dietary, or lifestyle parameters that could help explain the marked inter-individual variability in *S. salivarius* counts. Future studies should therefore recruit larger, age- and sex-balanced cohorts and incorporate data on oral hygiene habits, diet, medical history, and comorbidities to assess how these factors influence culture-based quantification. Expanding the protocol to include pediatric, elderly, and medically vulnerable groups will also be essential to confirm its robustness across diverse settings.

Regarding repeatability and agreement, duplicate plating was included in all assays to improve reproducibility and minimize counting errors. However, we did not perform formal statistical analyses such as intraclass correlation coefficients (ICC) or per-sample coefficients of variation (CV). As such, the protocol should be regarded as a semi-quantitative tool rather than a fully validated analytical method. We acknowledge this as a limitation and identify it as an important direction for future work aimed at enhancing statistical robustness and quantifying inter- and intra-assay variability.

To strengthen the protocol, future work should standardize sample handling, explore refined storage protocols, and aim to test the method across broader and more diverse populations. Future studies should also emphasize longitudinal monitoring of *S. salivarius* abundance in both pediatric and adult populations. Integrating culture-based quantification with host immune profiling and systemic biomarkers in longitudinal frameworks could yield a more holistic understanding of the role of oral commensals in health and disease.

We therefore plan a stratified follow-up study including pediatric, elderly and medically vulnerable cohorts (e.g., congenital heart disease, immunocompromised patients), with larger sample sizes (target ≥50–100 per subgroup), longitudinal sampling, and parallel molecular assays (qPCR/dPCR). This forthcoming work will formally evaluate analytical performance (repeatability, ICC, coefficient of variation) and enable establishment of age- and condition-specific reference ranges.

## 5. Conclusions

In conclusion, this study provides an accessible and low-cost 48 h culture-based protocol with MALDI-TOF/VITEK confirmatory identification for reliable quantification of *Streptococcus salivarius* in human saliva. Future work will focus on multi-site reproducibility, testing across broader demographic groups, and open dissemination of the protocol and dataset to facilitate large-scale adoption.

## Figures and Tables

**Table 1:** Calculation formula for CFU/mL.

Dilution	Colony Count Plate 1	Colony Count Plate 2	Average Colony Count	CFU/mL
10^−5^	x	y	T = (x + y)/2	T × 10^5^
10^−6^	n	m	O = (n + m)/2	O × 10^6^
10^−7^	z	w	V = (z + w)/2	V × 10^7^
			Average CFU/mL	(T + O + V)/3

**Table 2:** *Streptococcus salivarius* count in 18 saliva samples after 24 h and 48 h of incubation.

Sample No.	CFU/mL × 10^5^ (24 h)	CFU/mL × 10^5^ (48 h)
1	5.5	5.7
2	45.8	46
3	86.6	79.5
4	82	82.5
5	107.5	137
6	148.5	152.5
7	170	182.5
8	193.3	195
9	237.5	237.5
10	439.6	466
11	450.3	525
12	930	932.5
13	947.5	972.5
14	1152.5	1227.5
15	1257.5	1297.5
16	1262.5	1302.5
17	2090	2137.5
18	5200	6050

**Table 3:** CFU/mL of *Streptococcus salivarius* after 24 h storage at room temperature (RT) and 4 °C.

Initial CFU/mL × 10^5^	After 24 h at RT	After 24 h at 4 °C
1302.5	1467.5	not tested
932.5	1442.5	not tested
137	155	not tested
46	9	not tested
182.5	not tested	1112.5
79.5	not tested	0
237.5	not tested	335
5.75	not tested	11

**Table 4:** Comparative overview of the major methods for detection of *S. salivarius*.

Method	Sensitivity	Time to Results	Equipment/Cost	Output	Applicability
qPCR	High (10–100 genome copies)	3–5 h	High cost, requires thermocycler	Relative or absolute DNA copies	Clinical diagnostics, research
dPCR	Very high (1–10 copies)	5–6 h	Very high cost, specialized platform	Absolute DNA copies	Biomarker validation, precision diagnostics
LAMP	Moderate–high	1–2 h	Moderate; isothermal device needed	Presence/absence or relative quantification	Field studies, forensic identification
Culture-based (this study)	Moderate (10^3^–10^4^ CFU/mL)	48 h	Low; standard lab equipment	Viable CFU/mL	Routine labs, resource-limited settings

## Data Availability

The original contributions presented in this study are included in the article. Further inquiries can be directed to the corresponding author.

## Abbreviations

The following abbreviations are used in this manuscript:

*S. salivarius*	*Streptococcus salivarius*
CFU	colony forming unit
PBS	phosphate-buffered saline
MSA	Mitis-*Salivarius* Agar
